# Changes in the Carotenoids of *Zamia dressleri* Leaves during Development

**DOI:** 10.3390/plants13091251

**Published:** 2024-04-30

**Authors:** Enrique Murillo, Veronika Nagy, Dania Menchaca, József Deli, Attila Agócs

**Affiliations:** 1 Department of Biochemistry, Faculty of Exact Natural Sciences and Technology, University of Panama, Panama City 0824, Panama; 2 Department of Biochemistry and Medical Chemistry, Medical School, University of Pécs, Szigeti út 12, H-7624 Pécs, Hungary; vera.nagy@aok.pte.hu (V.N.); jozsef.deli@aok.pte.hu (J.D.); 3 Department of Pharmacognosy, Faculty of Pharmacy, University of Pécs, Rókus u. 2., H-7624 Pécs, Hungary

**Keywords:** cycads, *Zamia* species, carotenoid composition, HPLC, capsanthin–capsorubin synthase

## Abstract

It has been observed that the leaves of some Zamia species undergo a kind of “reverse ripening”; that is, they change from their original brown color to green during development. We assumed that this strange color change was due to the change in carotenoid composition, so we followed the changes for several weeks. The detailed carotenoid composition and content at different stages of development of the leaves was determined with HPLC-DAD focusing on the changes in red and yellow carotenoids. The total and relative amounts of red and yellow carotenoids were determined simultaneously from one measurement from a saponified and/or unsaponified extract. At the beginning of development, the concentration of red carotenoids was higher than that of the yellow ones; it decreased drastically until 22 days and continued to decrease slowly until they completely disappeared. The concentration of yellow carotenoids decreased at the beginning as well, but after 22 days it started to increase. The amount of red carotenoids started to decrease when the leaflet stopped growing. Lutein is the main component in old leaflets, which is not a red carotenoid precursor. Red carotenoids can always be found in their esterified form in the leaves. These findings support the hypothesis that red and yellow carotenoid accumulation are independent and probably have different functions in the leaflet. The strange color change was explained based on the compartmentalization of red and yellow carotenoids and on the changing activity of the enzyme capsanthin–capsorubin synthase responsible for the synthesis of red carotenoids capsorubin and capsanthin.

## 1. Introduction

**Figure 1:**
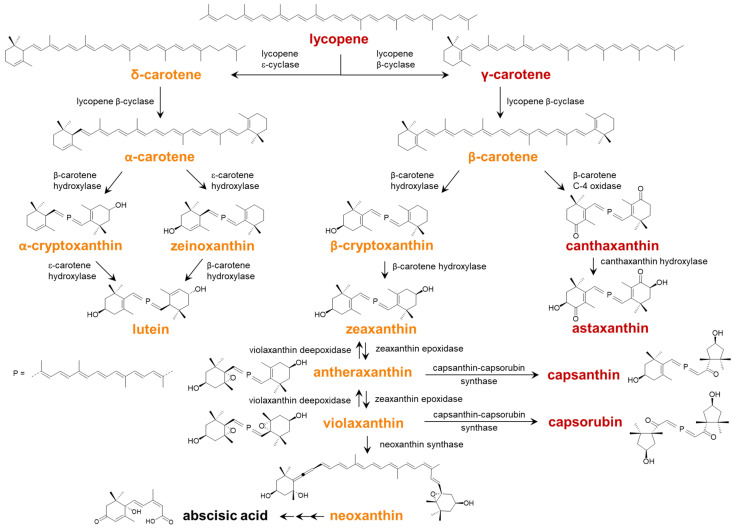
Main routes for biosynthesis of carotenoids [1]. Names are in yellow for “yellow carotenoids” (λ_max_ < 460 nm) and are in red for “red carotenoids” (λ_max_ > 470 nm). Also, see the text for definitions.

Carotenoids are ubiquitous isoprenoid pigments found in all living organisms from bacteria to humans. The biosynthesis of carotenoids in plants, fungi, bacteria and algae follows the mevalonate pathway yielding geranyl–geranyl pyrophosphate, from which phytoene, the precursor of lycopene, is produced. Cyclisation and subsequent hydroxylation of lycopene give xanthophylls, which can be further oxidized to epoxy carotenoids. The interconversion of violaxanthin to zeaxanthin via antheraxanthin was observed in the xanthophyll cycle involved in the photoprotection of plant chloroplasts. Antheraxanthin and violaxanthin are known to be directly converted to the red κ-carotenoids capsanthin and capsorubin, respectively, by the enzyme capsanthin–capsorubin synthase (Figure 1) [1]. Capsanthin and capsorubin are generally not found in the chlorophyll-containing tissues of higher plants and were thought to be unique to peppers (*Capsicum annuum*), but some tropical plants such as *Zamia dressleri*, jipi-japa (*Carludovica palmata*) or papua fruit (*Pandanus conoideus*) proved to contain them in high concentrations as well [2]. The relatively rare cyclopentane ring (κ-ring) is formed from the 3-hydroxy-5,6-epoxy-β-rings by capsanthin–capsorubin synthase, which was isolated from the chromoplasts of peppers. Actually, its amino acid sequence, and also its function, resembles that of the lycopene cyclase that produces β-rings [3].

Although many characteristics are known of the enzymes involved in their biosynthesis, the regulation pathways are yet to be elaborated in sufficient detail. In general, the central role of carotenoids in plant development and adaptation suggests that their synthesis is coordinated with other developmental processes such as plastid formation, flower formation and fruit development. These involve many enzymes that probably have more than one types of regulation at both the transcriptional and post-transcriptional levels. Chloroplasts and chromoplasts sequestrate carotenoids differently, thus the sequestration of carotenoids can be a type of regulation. In chloroplasts, the carotenoids become part of the light-harvesting machinery and the unbound carotenoids are associated with specific proteins [4]. In the chromoplasts of peppers, the carotenoids are esterified and bound to the fibrillin protein as fibrils. The esterification of carotenoids appears to be an effective mechanism used by a lot of flowers to produce high levels of them [5].

In photosynthesis, carotenoids act as light-collecting pigments and protectors against photo-oxidation [6]. Apart from their role in photosynthesis, carotenoids are the precursors of some phytohormones and of compounds that act as signal molecules for control of development in plants and in responses to environmental stimuli [7].

In this work, we classify carotenoids according to their spectral properties; we will call “red carotenoids” the ones which possess higher absorption maxima (λ_max_ > 470 nm) in ethanol and “yellow carotenoids” those with lower absorption maxima (λ_max_ < 460 nm) that are normally present in the chloroplast. These designations—which are actually used in everyday lab jargon—have nothing to do with the actual color of the carotenoids, which vary in different matrices.

The leaves of higher plants usually contain β-carotene, lutein, violaxanthin and neoxanthin, with small amounts of other carotenoids, such as the yellow ones [8]. These carotenoids are found in chloroplasts linked to the thylakoid membranes, mainly in their non-esterified form [9,10]. In photosynthetic tissues, the color of the carotenoids is masked by the green color of the chlorophylls, since that is found in greater proportion [11]. However, in leaves with a high content of red ketocarotenoids, the presence of these is reflected in the color of the tissue. Czeczuga reported high amounts of adonixanthin and rhodoxanthin in gymnosperm leaves in autumn and winter [12]. The presence of novel seco-carotenoids has been reported in the red-brown leaves of the *Ceratozamia* species [13,14].

*Zamia* is a genus of cycads widely distributed between Mexico and Central and South America. Cycads belong to the clade of primitive gymnosperms, which constituted a quarter of terrestrial vegetation in the Mesozoic era (150 million years ago) [15]. In recent years, interest in learning about the biological aspects that have allowed the survival of these plants, even under adverse conditions, has intensified [16,17,18,19,20]. In Panama, twelve species of zamias have been identified, which are endemic (they only exist in Panama). Six of them are characterized by having red-brown leaves in the early stages of their development [21,22], that turn to green in older specimens. *Zamia dressleri* is one of these species, it grows in evergreen lowland forests, mainly in provinces such as Colón and Guna Yala (see map Supplementary S1). Its conservation status is threatened, and it is in danger of extinction. Recently, it has been shown that the color of the young leaves (red-brown) of *Z. dressleri* is due to the high content of capsorubin and capsanthin [23]. Sources of keto-carotenoids are of special interest as they exert much higher antioxidant potential than other carotenoids [24]. 

In this paper we evaluated the qualitative and quantitative changes of the carotenoids present in the leaflets of *Z. dressleri* during their development for five months until they reached their final color and size. A novel methodology was implemented, which allowed for the simultaneous determination of yellow and red carotenoids in many samples.

## 2. Results and Discussion

### 2.1. Carotenoids in Young and Old Leaflets

**Figure 2:**
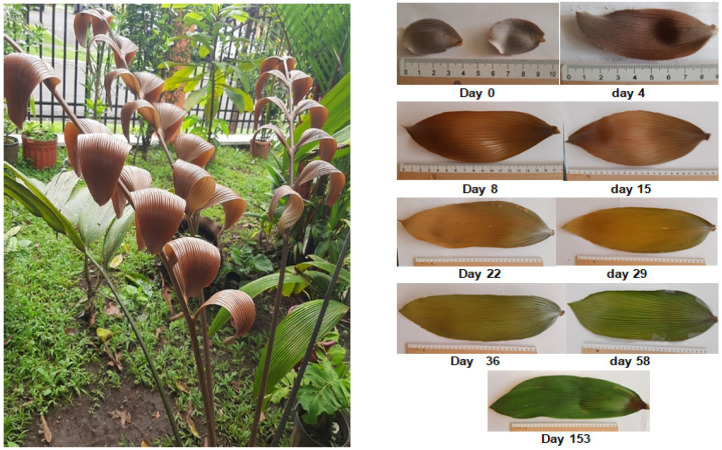
Changes in color and morphology of *Zamia dressleri* leaves.

In *Z. dressleri,* two leaves are usually born, each with six pairs of leaflets, although sometimes the number of new leaves can be three or four. Figure 2 shows a *Z. dressleri* plant with leaves and leaflets in the red-brown phase and the morphological changes they undergo during development.

In this study, the first leaflets were collected immediately after the separation of the pairs considering this point as t = 0 day. At this point the leaflets were deep red-brown in color and very soft in texture. During development, the intensity of the red-brown color gradually decreased, until it was replaced by the green, characteristic color of the leaves in active photosynthesis; in addition, the leaflets acquired a rigid texture, which made them brittle. 

**Figure 3:**
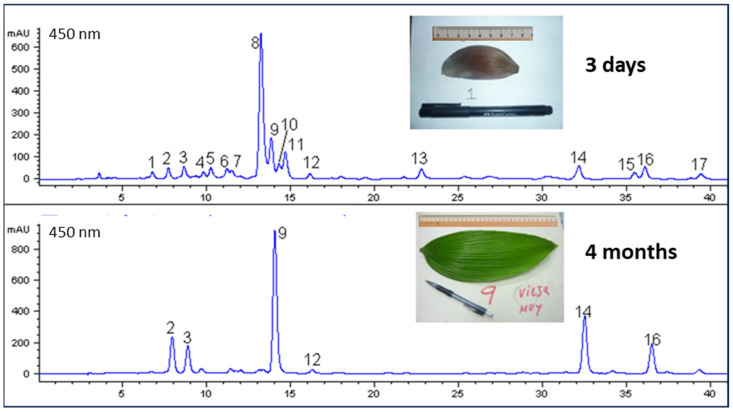
Carotenoids in young and old leaves of *Zamia dressleri* after saponification. 1: unknown; 2: neoxanthin (Y); 3: violaxanthin (Y); 4: 5;6-epoxycapsanthin (R); 5: *cis*-capsorubin (R); 6: antheraxanthin (Y); 7: mixture; 8: capsorubin (R); 9: lutein (Y); 10: unknown; 11: capsanthin (R); 12: zeaxanthin (Y); 13: cryptocapsin (R); 14: α-carotene (Y); 15: unknown; 16: β-carotene (Y); 17: unknown. (R) Red carotenoid; (Y) Yellow carotenoid.

The carotenoid composition was investigated by HPLC-DAD analysis of the saponified extracts in all stages. In Figure 3, it can be seen that, in the early collected leaflets (3 days), the main carotenoid was capsorubin, but otherwise they were mainly characterized by some other κ-carotenoids, such as capsanthin and their isomers. These are the red carotenoids, the carotenoids with a κ-end group (peaks 4, 5, 8, 11, 13), which possess a higher absorption maxima (λ_max_ > 470 nm) than the yellow carotenoids (λ_max_ < 460 nm) from the chloroplasts (main peaks 2, 3, 9, 14, 16).

We have recently demonstrated that leaflets contain 325 µg/g of capsorubin at t = 0 and 59 µg/g after 20 days [23]. Figure 3 shows that, when the leaflets turn green (after 4 months), capsorubin almost completely disappears and typical thylakoid carotenoids (yellow carotenoids, mainly lutein) predominate [8]. All the results of this study are presented on a fresh basis, although leaflet moisture varies from 95% at t = 0 to 70% after 148 days.

### 2.2. Changes in Red and Yellow Carotenoids during Development

At the beginning of development, the concentration of red carotenoids in leaves is higher than that of the yellow ones, but it decreases drastically until 22 days and continues to decrease slowly until it completely disappears (Table 1).

The concentration of yellow carotenoids decreases at the beginning, as well, but after 22 days it starts to increase. This turning point coincides with the decrease in the rate of change in the red ones.

This behavior may lead us to think that the decrease in the concentration of red carotenoids after 22 days is partly due to a decrease in their synthesis, since yellow carotenoids increase during this period. However, in old leaflets the main carotenoid is lutein, which is not a precursor of capsorubin or capsanthin. In the fruits of peppers, only zeaxanthin, the constitutional isomer of lutein, can be found and it is transformed during ripening to epoxides and κ-carotenoids, which are all esterified. On the other hand, the ratio of concentration for violaxanthin (direct capsorubin precursor) and lutein, which can be observed after 4-6 months (Figure 3), is typical of a green leaf. For this reason, the decrease in red carotenoids is probably mainly due to increased degradation. Perhaps the metabolism products of red carotenoids play some physiological role in these leaflets. This hypothesis needs further proof, of course, although it is known that some carotenoid metabolites act as hormones that regulate metabolism in plants [25]. In studies on the red-brown leaves of ceratozamia, it has also been found that the amount of keto-carotenoids (red) decreases during leaflet development until they disappear [26].

In the first few days, the growth of the leaflets was found to be very fast, increasing their weight about 20 times in 15 days and 30 times in 22 days in relation to t = 0 (Table 1). This phase of growth coincides with the rapid drop in the concentration of the red and yellow carotenoids. The decrease in carotenoid concentration is probably due to the fact that the rate of cell duplication in the leaflet is greater than the rate of carotenoid synthesis and accumulation. The absolute amounts of red and yellow carotenoids per leaflet presented in Table 1 demonstrate the changes together with the weights of the leaflet (Supplementary S2). It can be observed that the total amount of red carotenoids increases up to 22 days indicating that in this phase the rate of synthesis is greater than that of degradation. Another observation is that after 4 weeks the amount of red carotenoids started to decrease and the leaflet stops growing. This suggests that there may be some correlation between the content of red carotenoids and the development of the leaflet. The red carotenoids may promote growth, or their breakdown products may be signals to stop it, as carotenoids are known precursors of plant hormones [27]. There is no evidence yet that carotenoid cleavage dioxygenases (CCD) can use kappa carotenoids as a substrate [28].

The amounts of yellow carotenoids continued to increase and they decreased when leaflet turns very old and the leaflet became a “normal” green leaf which contains practically no κ-carotenoids. The accumulations of red and yellow carotenoids seem to be independent from each other and probably have different functions in the leaflet, which also suggests that they can be found in different compartments of the cell; it is possible that the red carotenoids are in chromoplasts as in paprika, but they definitely outside the thylakoid. The chromoplasts are known to exert special physiological functions that are independent from chloroplasts [29].

It may be interesting to see how the chlorophyll concentration changes over time. It was observed that, in the early stages of development, the amount of chlorophylls and carotenoids tends to decrease (Table 2, Supplementary S3). Then, the amounts of chlorophylls and yellow carotenoids increase, while the red carotenoids disappear. This behavior supports the role of yellow carotenoids as accessory pigments in photosynthesis, along with chlorophyll.

### 2.3. Changes in the Native Carotenoid Composition

**Figure 4:**
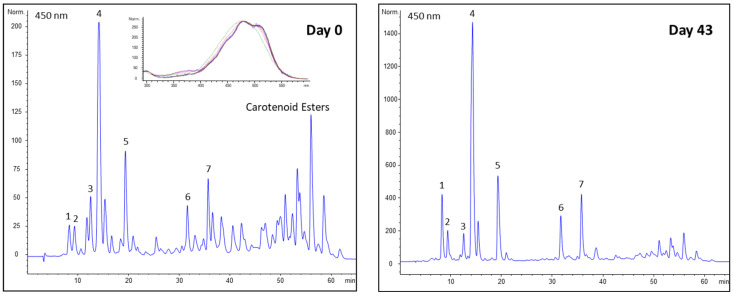
Changes in native carotenoid composition during development. 1: neoxanthin (Y); 2: violaxanthin (Y); 3: antheraxanthin (Y); 4: chlorophyll-*b* + lutein (Y); 5: chlorophyll-*a*; 6: α-carotene (Y); 7: β-carotene (Y). (R) Red carotenoid; (Y) Yellow carotenoid. Insert: superimposition of UV–vis spectra of carotenoid esters.

The esterification or non-esterification of the carotenoids may also possess important background information about whereabouts of the carotenoids in the leaflets; thus, the unsaponified extracts were also studied. Figure 4 shows that, at the beginning (t = 0), carotenoid esters (unnumbered peaks with high retention times) predominate, but they decrease during development (43 days).

When the UV–vis spectra of the carotenoid esters are superimposed, their maximum is around 480 nm in the region of the spectrum that corresponds to the red carotenoids (Supplementary S4). This is the first report on the presence of carotenoid esters in the red-brown leaves of plants, which contain hydroxylated red carotenoids. It is known that thylakoid carotenoids (yellow) are found in their non-esterified free form in chloroplasts [30]. Cardini et al. found that the keto carotenoids of the red-brown leaves of ceratozamia are located in the stroma of the chloroplasts [13]. Our observations also suggest that the red carotenoids do not occur together with the yellow ones in chloroplasts. An interesting fact is that in the young leaflets of *Zamia* the red carotenoid esters accumulate without the esters of their yellow precursors being present at all. There may be at least two reasons for this finding: red carotenoid precursors (violaxanthin) may migrate from the thylakoid membrane to the stroma or another organelle, where the enzyme capsantin–capsorubin synthase (CSS) catalyzes capsorubin and capsanthin synthesis and then they accumulate as esters. As we mentioned earlier, esterification is a common strategy in plants for the storage and accumulation of carotenoids eg. in petals [5]. Another alternative is that in the organelle the CSS activity is so high that it does not allow for the accumulation of violaxanthin esters as they are present in very minor amounts if at all in the extract.

### 2.4. Changes in Zamia-specific Carotenoids

In order to evaluate the changes in carotenoid composition, we also studied *Zamia neurophylidia*, which is one of the endemic *Zamia* species in Panama whose leaves are always green, as is the case in most plants. As shown in Table 2, which compares the leaf carotenoids of this species with those of *Zamia dressleri*, in the case of *Z. neurophylidia* there is less change in carotenoid composition during development. In *Z. dessleri,* at the beginning, capsorubin is the main carotenoid followed by lutein and capsanthin. The concentration of capsorubin decreases with time until it is not detectable on day 148. If the thylakoid yellow carotenoids are compared between the green leaflets of *Z. dressleri* and *Z. neurophylidia* (148 days), it is observed that they have a similar composition. If only the yellow carotenoids are considered, there is similar amount during development and similar changes occur in green or red-brown *Zamia* species, so *Z. dressleri* is rather ordinary in that sense. One change that must be noted is that, in both species, the α/β carotene ratio increases during development, a behavior that has been reported in ceratozamia [25].

**Table 2:** Changes in leaf carotenoids (%) of *Zamia dressleri* and *Zamia neurophylidia* during development.

Compound Days	*Zamia dressleri*				*Zamia neurophylidia*	
	0	22	43	148	0	148
Neoxanthin (Y)	2.6	6.7	9.4	10.9	15.4	11.9
Violaxanthin (Y)	3.1	4.1	4.3	7.9	4.7	9.4
Capsoneoxanthin (R)	1.6	2.3	1.6	nd	nd	nd
*cis*-Capsorubin a (R)	2.9	2.6	1.5	nd	nd	nd
Antheraxanthin (Y)	2.8	1.9	1.6	1.7	9.5	1.5
Capsorubin (R)	42.6	28.2	13.1	nd	nd	nd
Lutein (Y)	11.5	7.5	35.0	41.7	41.0	44.0
*cis*-Capsorubin b (R)	3.9	4.4	1.9	nd	nd	nd
Capsanthin (R)	7.9	8.9	5.2	nd	nd	nd
Zeaxanthin (Y)	1.4	0.5	0.9	1.2	4.0	1.7
Cryptocapsin (R)	3.3	1.0	nd	nd	nd	nd
α-Carotene (Y)	4.6	3.1	5.2	19.0	5.0	17.0
β-Carotene (Y)	4.2	6.2	11.5	9.5	18.4	10.0
*cis*-β-Carotene (Y)	2.0	0.9	1.4	1.3	1.9	1.1
Others * (R) + (Y)	5.6	11.7	7.4	6.8	1.1	3.4
**Total (µg/g)**	**646**	**148**	**149**	**143**	**95**	**129**
Chlorophyll *a*	316	160 **	485	646		
Chlorophyll *b*	165	87 **	179	332		

(R) Red carotenoid; (Y) Yellow carotenoid; nd means <0.05%; * Carotenoids detected but not identified; ** Chlorophylls were determined on day 15, see also Supplementary S2.

## 3. Materials and Methods

### 3.1. Sample Collection

The leaflets of *Zamia dressleri* and *Zamia neurophylidia* were collected from the International Cycad Garden located on the central campus of the University of Panamá. The aging and growth of six leaves from three plants were followed.

### 3.2. Extraction and Saponification of Carotenoids

The freshly collected leaflets were weighed, separating a portion for moisture determination and another for carotenoid analysis. The analyses were carried out on the fresh samples on the day of collection, essentially following the procedure recommended by Britton [31]. Half of a leaflet was accurately weighed, placed in a porcelain mortar, homogenized with 1% sodium bicarbonate and extracted with acetone until no more color could be observed. The extract was diluted with the same amount of diethyl ether/hexane (1:1), washed with water, dried with Na_2_SO_4_, filtered and the solvents were evaporated under vacuum at 35 °C (rotary evaporator). An aliquot of this unsaponified extract was used to determine the native carotenoid composition by HPLC-DAD. The determination is based on the previous LC-MS identification of the carotenoids [23].

The crude extract was dissolved in diethyl ether and saponified adding an equal volume of 5% methanolic KOH and allowing it to settle for two hours. A further amount of ether was added, the mixture was washed with water to remove excess of the base and the phase containing the carotenoids, after drying on Na_2_SO_4_, was evaporated in vacuum. The saponified extract was stored dissolved in hexane at −20 °C under nitrogen atmosphere.

### 3.3. Simultaneous Determination of Red and Yellow Carotenoids

Considering that the determination of total carotenoids, by reading the absorbance at 450 nm and expressing the results as β-carotene, underestimates the content of red carotenoids, we worked out a simultaneous determination of red and yellow carotenoids applying the principle suggested by Harvey [32]. From the total volume of the extract the amount of total carotenoids was determined and, considering the mass of the leaflet, their concentration in µg/g could be evaluated. A methodology that uses both the A1% values of lutein (yellow) and capsorubin (red) has not been reported so far. A1% is the absorbance of a solution of 1 g/100 mL concentration at a given wavelength.

Using authentic standards, the A1% values for lutein and capsorubin were determined at 445 and 510 nm in ethanol, as representatives of the yellow and red carotenoids. The lutein standard was isolated from pumpkin (*Cucurbita maxima*) and the capsorubin standard from red pepper (*Capsicuum annuum*). The results are presented in Table 3.

The concentration of red and yellow carotenoids was determined by reading the absorbance of the saponified extract at 445 and 510 nm (A_445_ and A_445_, respectively), and then using Equations (1) and (2).

A_445_ = 1353.3 c_cap_ + 2550 c_lut_ (1)

A_510_ = 2013.6 c_cap_ + 82.6 c_lut_ (2)

c_cap_ = concentration of capsorubin (red carotenoids);
c_lut_ = concentration of lutein (yellow carotenoids).
Solution for c_cap_ using Equation (1):

(3) $$c_{\mathrm{cap}\;}=\frac{A_{445}-2550c_{\mathrm{lut}\;}}{1353.3}(g/100\;\mathrm{mL})$$

Using the above expression for c_cap_ in Equation (2):

A_510_ = 2013.6 (A_445_ − 2550 c_lut_)/1353.3 + 82.6 c_lut_ (4)

And finally, solving for c_lut_:

(5) $$c_{\mathrm{lut}\;}=\frac{1.49A_{445}-A_{510}}{3716.9}(g/100\;\mathrm{mL})$$

With the absorbances at 445 and 510 nm and the Equation (5), the concentration of yellow carotenoids (c_lut_) can be determined. Then, from Equation (3) the concentration of red carotenoids (c_cap_) can be calculated. Applying the dilution factors and knowing the total mass of the extract, the total amount of red and yellow carotenoids can be determined in the usual µg (carotenoid)/g (extract) unit.

### 3.4. Analysis of the Carotenoids by HPLC-DAD

Individual red and yellow carotenoids were quantified based on the total amount of red and yellow carotenoids spectrophotometrically determined in Section 3.3 and on their correspondent HPLC integrated area fraction.

All solvents used in the high-performance liquid chromatography analysis were of HPLC grade. For the separation of carotenoids Hewlett Packard 1050 liquid chromatograph equipped with a Diode Array Detector (HPLC-DAD) was used. The chromatograms were captured at 450 nm and the data were processed with the ChemStation program. The separation was carried out on an endcapped C_30_ column (250 × 4.6 mm i.d.; YMC C30, 3 μm, YMC Europe GmbH, Dinslaken, Germany). Eluents: (A) MeOH: MTBE: H_2_O = 81:15:4 *v/v*%; (B) MeOH: MTBE: H_2_O = 6:90:4 *v*/*v*%. The chromatography was performed in a linear gradient from 100 % A eluent to 50 % B mixture in 45 min, with 1.00 mL/min flow rate at 25 °C.

### 3.5. Identification of the Peaks

The carotenoids were identified using the following data: elution order on the C_30_ HPLC column, spiking with authentic standards, UV–vis spectrum (λ_max_, spectral fine structure (%III/II), cis peak intensity (%AB/II) and mass spectrum (molecular ion and fragments) compared to standards and data available in the literature [33]. Authentic samples were taken from our collection. Spiking was carried out one by one for each component in cases in which the identification was ambiguous. The amount of standard was comparable with the amount of the unknown carotenoid in the plant extract. Purity was assessed based on the areas in the HPLC chromatograms at 450 nm.

## 4. Conclusions

Some *Zamia* species such as *Zamia dressleri* have the strange behavior that the color of their leaves change from brown to green rather than the other way around, which is the usual manner for deciduous plants. We proved that this is a consequence of the high content of red carotenoids (especially capsorubin) in the young leaves, which degrades—supposedly to plant hormones—over time. During that time, in the thylakoid membrane of the chloroplasts, the usual carotenoids lutein and epoxy carotenoids that are needed for the photosynthetic machinery are synthetized.

We postulate that the initial red carotenoids cannot be in the thylakoid as they are all esterified, whereas yellow carotenoids are not esterified at all. The high activity of CCS outside the thylakoid (maybe in a chromoplast) may be responsible for the presence of red carotenoids esters and for the absence of the precursor yellow esters. As the red compounds break down, the green color of the chlorophylls becomes visible again. However, the relative amount of red carotenoids increases during the first week, so they or their metabolic derivatives may have a signaling function or play a role in leaf growth. The amount of yellow (thylakoid) carotenoids in the leaves is similar to that in other species such as *Z. neurophylidia*, which occur in green leaves all the time, which means that chloroplasts of *Z. dressleri* show a conventional behavior.

In summary, the high red carotenoid content in the initial growth stage of *Zamia dressleri* is an “evolutionary surplus” shared by only a few similar species. Considering the ancient origin of the entire cycad family, this behavior may be a remnant of some evolutionary step that other species have grown out of or it may indicate that they have found other ways to exert the same function as the red carotenoids.

*Z. dressleri* is an endangered species whose last habitat may be in Panamá. The brown leaves are an excellent natural source of the rare carotenoid capsorubin which is a very strong antioxidant and has a plethora of advantageous medicinal effects. Maybe its cultivation for capsorubin could solve this problem and save this ancient species from extinction.

## Figures and Tables

**Table 1:** Amount of carotenoids (±standard deviation) in the leaflets of *Z. dressleri*. (n = 5).

Days	Mass of Leaflet g	Yellow µg (Total)	Yellow µg/g Leaflet	Red µg (Total)	Red µg/g Leaflet	Days
0	0.5 ± 0.1	77 ± 14	169 ± 6	218 ± 32	477 ± 10	0
4	1.7 ± 0.4	219 ± 48	131 ± 8	516 ± 69	309 ± 15	4
8	4.0 ± 0.6	369 ± 33	95 ± 6	726 ± 76	186 ± 7	8
15	9.5 ± 1.0	681 ± 98	71 ± 2	1023 ± 113	108 ± 7	15
22	15.1 ± 0.7	1006 ± 94	67 + 3	1233 ± 101	81 ± 2	22
29	18.8 ± 1.2	1318 ± 129	70 ± 2	1140 ± 118	61 ± 2	29
36	19.1 ± 1.0	1751 ± 188	91 ± 4	904 ± 90	48 ± 2	36
43	18.9 ± 0.9	2091 ± 84	111 ± 3	713 ± 59	38 ± 1	43
58	18.9 ± 1.1	2332 ± 123	123 ± 3	568 ± 56	30 ± 2	58
73	19.4 ± 1.4	2660 ± 85	138 ± 3	497 ± 30	26 ± 1	73
88	17.7 ± 0.8	2713 ± 152	153 ± 4	320 ± 67	18 ± 2	88
118	17.4 ± 0.7	2865 ± 310	172 ± 4	164 ± 34	9 ± 2	118
148	17.0 ± 0.5	2488 ± 165	143 ± 5	51 ± 7	3 ± 1	148
178	18.2 ± 0.5	1911 ± 128	105 ± 6	nd	nd	178

nd: not detected.

**Table 3:** A1% values for lutein and capsorubin at 445 and 510 nm in ethanol.

Compound	A1% at 445 nm	A1% at 510 nm
Lutein	2550.0	82.6
Capsorubin	1353.3	2013.6

## Data Availability

Data are contained within the article and Supplementary Materials.

## Supplementary Materials

The following supporting information can be downloaded at: https://www.mdpi.com/article/10.3390/plants13091251/s1, S1. Habitat of Zamia dressleri, S2. Relative and absolute changes in the carotenoids of Zamia dressleri leaves, S3. Chlorophylls and carotenoids in the leaflets of Zamia dressleri, S4. Superposition of Red and Yellow Carotenoid Spectra.
