# Correction: Murillo et al. Changes in the Carotenoids of *Zamia dressleri* Leaves during Development. *Plants* 2024, *13*, 1251

**DOI:** 10.3390/plants15050698

**Published:** 2026-02-26

**Authors:** Enrique Murillo, Veronika Nagy, Dania Menchaca, József Deli, Attila Agócs

**Affiliations:** 1 Department of Biochemistry, Faculty of Exact Natural Sciences and Technology, University of Panama, Panama City 0824, Panama; ainath_27@hotmail.com; 2 Department of Biochemistry and Medical Chemistry, Medical School, University of Pécs, Szigeti út 12, H-7624 Pécs, Hungary; vera.nagy@aok.pte.hu (V.N.); jozsef.deli@aok.pte.hu (J.D.); 3 Department of Pharmacognosy, Faculty of Pharmacy, University of Pécs, Rókus u. 2., H-7624 Pécs, Hungary

There was an error in the original publication [1]. A correction has been made to Section 3.3. Simultaneous Determination of Red and Yellow Carotenoids, Equation (5):

(5) $$c_{lut}=\frac{1.49A_{445}\;-A_{510}}{3716.9}\;(g/100\;mL)$$

The authors state that the scientific conclusions are unaffected. This correction was approved by the Academic Editor. The original publication has also been updated.
