# Supplementary material for: Changes in the Carotenoids of Zamia dressleri Leaves during Development
Source: Plants (Basel). 2024 Apr 30;13(9):1251. doi: 10.3390/plants13091251 (PMC11085121; doi:10.3390/plants13091251)

Supplementary Material

Changes in the Carotenoids of *Zamia dressleri* Leaves During Development

Enrique Murillo*[,a], Veronika Nagy[b], József Deli[b,c], Attila Agócs*[,b]

[a]Department of Biochemistry, Faculty of Exact Natural Sciences and Technology, University of Panama, Panama City, Panama
[b]Department of Biochemistry and Medical Chemistry, Medical School, University of Pécs, Szigeti út 12, 7624, Pécs, Hungary
[c]Department of Pharmacognosy, Faculty of Pharmacy, University of Pécs, Rókus u. 2., H-7624 Pécs, Hungary

*Corresponding authors: emurillo29@hotmail.com, phone: (507) 62879252
attila.agocs@aok.pte.hu, phone: +36 72 536 001/31864 ext.

S1. Habitat of *Zamia dressleri*

The main habitat is the rain forest of Panama, specifically in the coastal provinces towards the Caribbean Sea (Colón and Guna Yala).

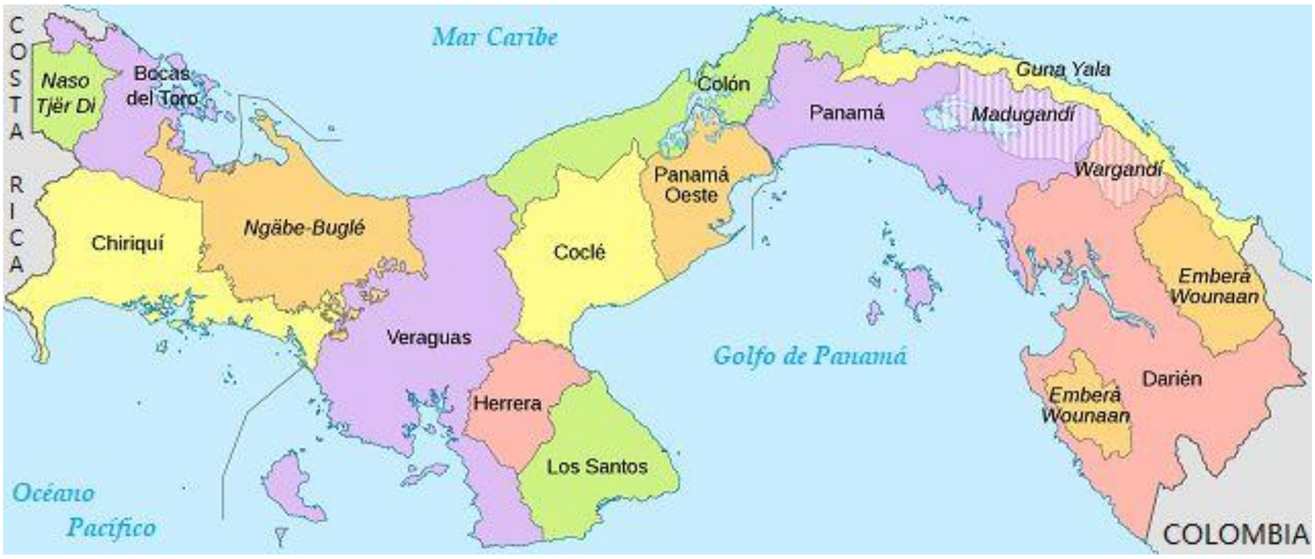

S2. Relative and absolute changes in the carotenoids of *Zamia dressleri* leaves

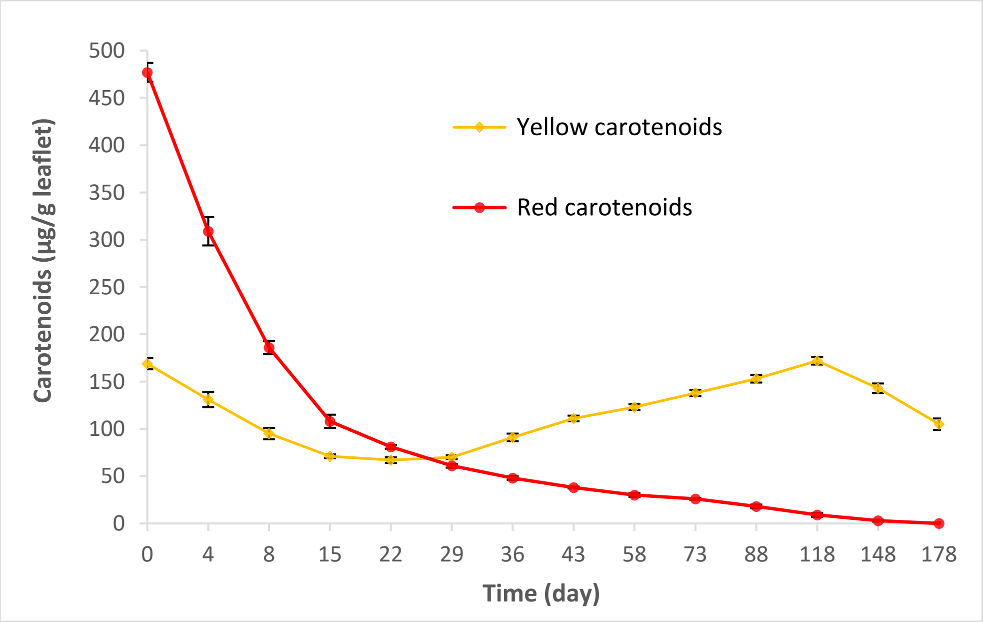

Figure S2.a. Changes in the concentration of the carotenoids in *Z. dressleri*. Each point in the graph corresponds to the average of five parallel determinations; the standard deviation data for each point is presented in Table 1.

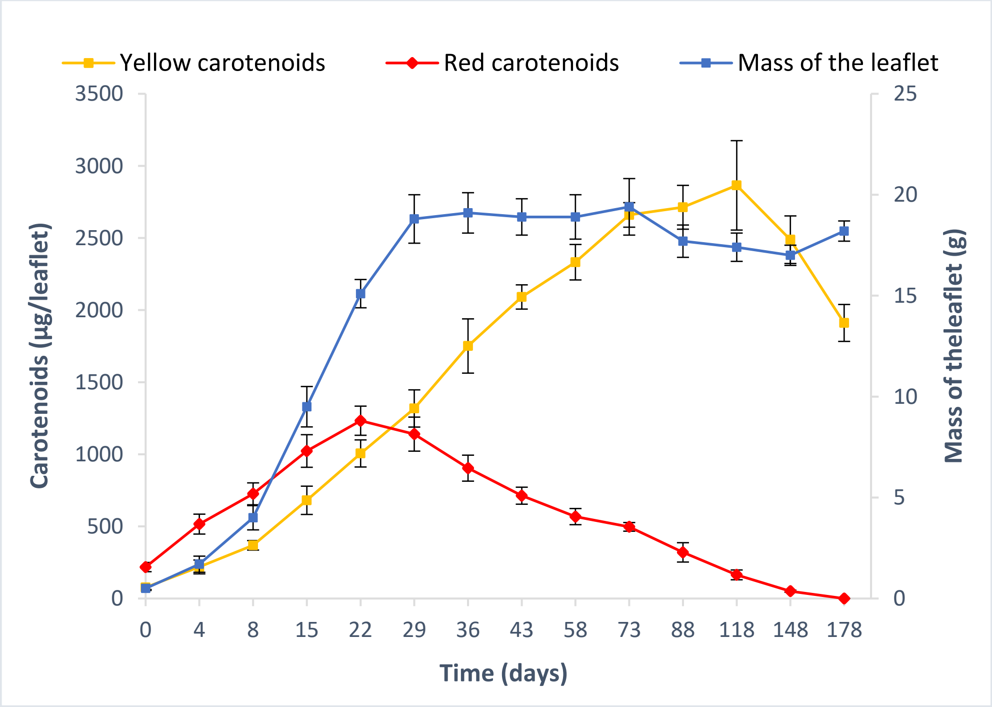

Figure S2.b. Effect of aging on the amounts of carotenoids in Zamia dressleri leaflets, independent of weight of the leaflet. The standard deviation data for each point is presented in Table 1 in the main text.

S3. Chlorophylls and carotenoids in the leaflets of *Zamia dressleri*

Days	Chlorophylls (μg/g leaflet)		Carotenoids (μg/g leaflet)	
	a	*b*	Yellow	Red
0	316	165	150	440
7	195	100	101	180
15	160	87	70	121
43	485	179	144	52
148	646	332	156	0

No statistical information is presented, because these are preliminary data, obtained from one leaflet and only the chlorophylls and carotenoids of one sample were analyzed.

Approximately 2 grams of fresh leaflet, weighed accurately, were extracted with several portions of acetone, until no more color was obtained. The extracts were transferred to diethyl ether and the acetone was removed by several washes with water. Residual water from the ether was removed using anhydrous sodium sulfate.
The concentration of chlorophylls a and b was determined in ethereal extracts of the leaflets, using the Smith and Benitez equations (Ec1 and Ec2), described by Jeana Gross in Pigments in Vegetables: Chlorophylls and Carotenoids, 1991, Springer Science + Business Media, New York .

Ec1 Chlorophyll *a* (mg/L) = $10.1\ A_{662} – 1.01\ A_{644}$
Ec2 Chlorophyll *b* (mg/L) = $16.4\ A_{644}$ -- $2.57 A_{662}$

The concentration of yellow and red carotenoids was determined in an aliquot of the ethereal extract, evaporated and dissolved in methanol, using the equations described in the main text.

S4. Superposition of Red and Yellow Carotenoid Spectra

Figure S3. Superposition of the spectra of the carotenoid esters in the non-saponified extract

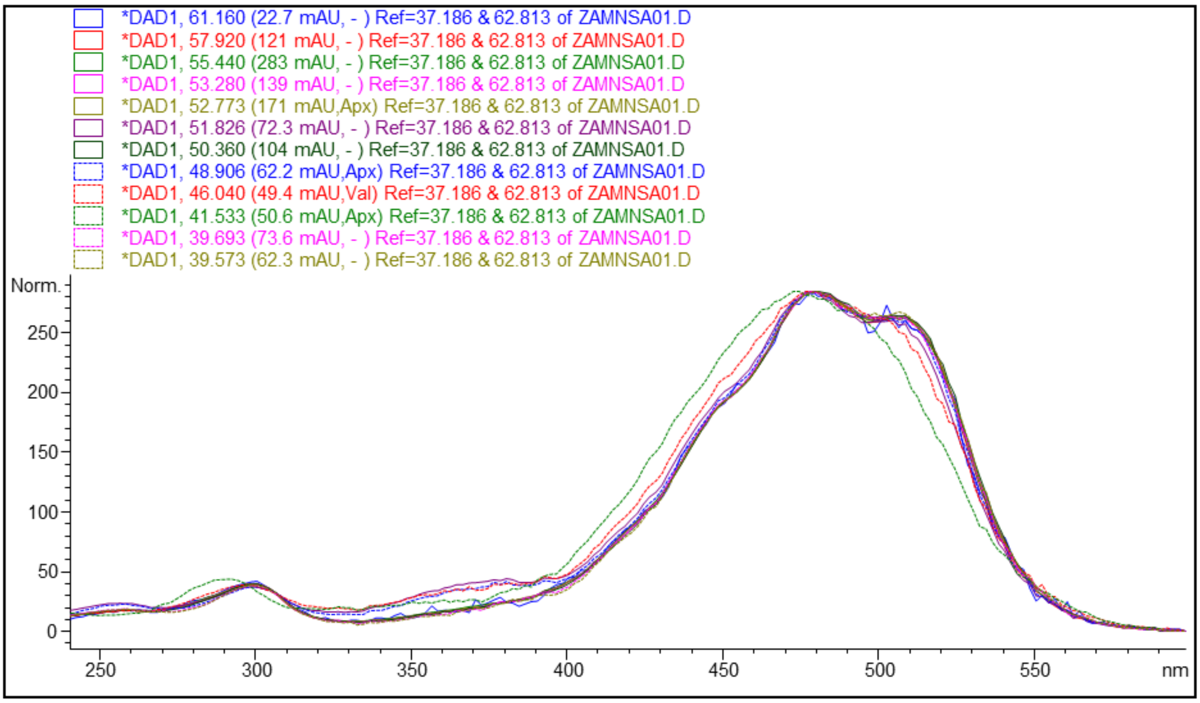

Figure S4. Superposition of the spectra of the yellow carotenoids in the saponified extract

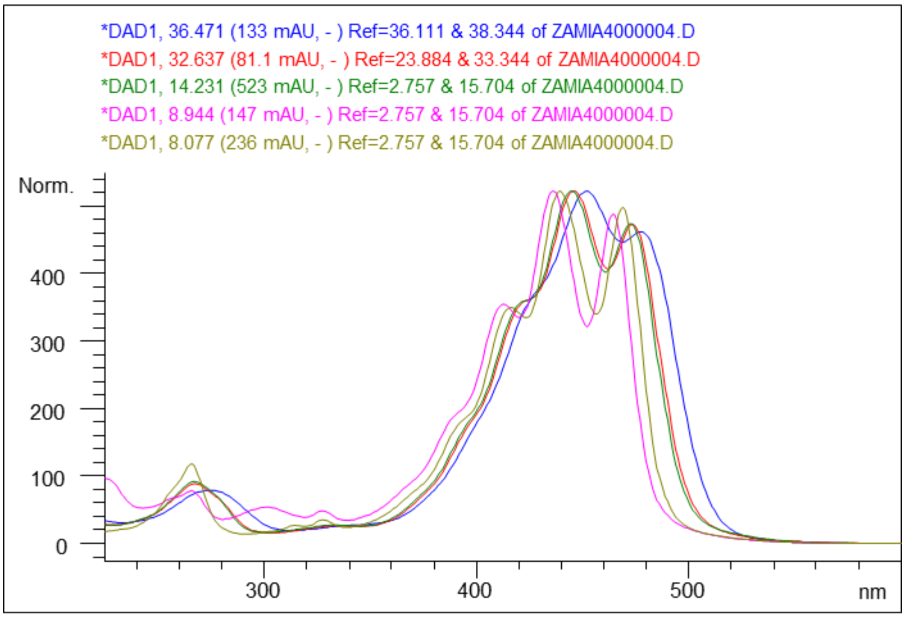

Figure S5. Superposition of the spectra of the red carotenoids in the saponified extract

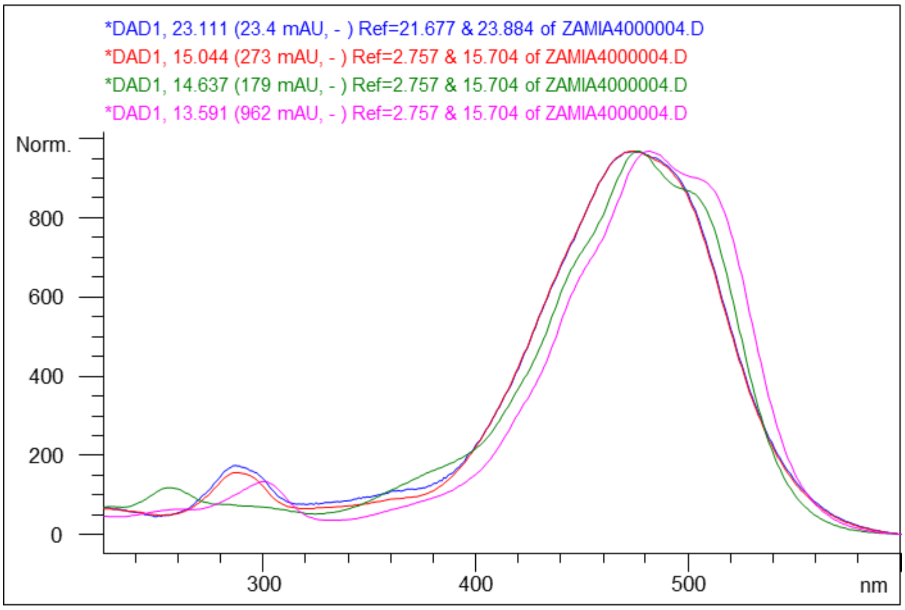

Supplement: Supplementary file 1 [file plants-13-01251-s001.zip › plants-2956601-supplementary.pdf]
